# Synaptic Plasticity is Altered by Treatment with Pharmacological Levels of Retinoic Acid Acting Nongenomically However Endogenous Retinoic Acid has not been shown to have Nongenomic Activity

**Published:** 2022-01-28

**Authors:** Gregg Duester

**Affiliations:** Development, Aging, and Regeneration Program, Sanford Burnham Prebys Medical Discovery Institute, USA

## Abstract

Retinoic acid (RA) is the active form of vitamin A that functions as a ligand for nuclear RA receptors that directly bind genomic control regions to regulate gene expression. However, some studies have suggested that RA may have nongenomic effects outside of the nucleus, particularly with regard to synaptic plasticity. Recent results demonstrate that treatment with pharmacological levels of RA can alter synaptic plasticity which may be useful to treat neurological diseases. However, these results and those reported by others have not shown that endogenous RA is normally required for synaptic plasticity (or any other nongenomic effect) as there are no reports of genetic loss of function studies that remove endogenous RA in adult brain. The implication is that pharmacological levels of RA result in nongenomic effects, some of which may be helpful to treat certain diseases but in other cases this may cause unwanted side effects.

## Pharmacological Levels of RA Alter Synaptic Plasticity

Several decades of studies have shown that RA serves as a ligand for nuclear RA receptors that directly bind genomic control regions known as RA response elements to regulate gene expression during development and adult homeostasis [1,2]. Lenz et al. report that RA treatment affects forebrain cortical synaptic plasticity which modulates synaptic transmission to effectively respond to specific stimuli; specifically, they report that this effect occurs in the dorsal hippocampus but not ventral hippocampus and requires synaptopodin [3]. The method Lenz et al. used to determine RA function was to treat mice with 10 mg/kg RA. This dose of RA results in micromolar concentrations of RA in mouse tissues which is a teratogenic dose [4]; RA is normally present in the nanomolar range in brain and other tissues [5]. Such high levels of RA cannot be used to determine the normal function of RA due to unwanted side effects. In fact, one cannot determine the normal in vivo functions of RA by adding RA. Instead, one must take away RA preferably using RA genetic loss of function studies in vivo to be certain that RA is required for any proposed function [6,7].

The article by Lenz et al. follows up on a series of other articles suggesting that RA controls synaptic plasticity in a nongenomic fashion using a mechanism that does not involve regulation of gene expression by nuclear RA receptors, but instead involves cytoplasmic RA receptors that control mRNA translation or perhaps other nongenomic processes [7-10]. However, although these previous articles suggest that endogenous RA normally controls synaptic plasticity, none report a genetic loss of function study to remove endogenous RA to see if it is required for synaptic plasticity via any mechanism. In order to do this in brain is difficult as several enzymes may participate in RA synthesis. However, it has been possible to eliminate RA synthesis in mouse fetal brain using knockouts of the RA-generating enzymes encoded by Aldh1a2 or Aldh1a3 [11]; RA production has also been successfully eliminated using a triple conditional knockout of Aldh1a1, Aldh1a2, and Aldh1a3 in adult mouse cornea [12] and ovary or testis [13-15]. Although sometimes very difficult, genetic loss of function studies are needed to determine the function of any gene, protein, or in this case a molecule such as RA.

## Summary

As it stands now, Lenz et al. have provided convincing evidence that pharmacological levels of RA can alter synaptic plasticity in a synaptopodin dependent fashion; they did not address whether endogenous RA normally controls synaptic plasticity (Lenz et al., 2021). Such information may be useful to explore treatment options for neurological disease. However, in order for the reader to be able to fully understand how these studies relate to normal RA signaling, it is useful to point out that future studies are needed to determine whether endogenous RA controls synaptic plasticity. In order to accomplish this goal, one would need to determine whether endogenous RA is present in the relevant tissues, determine where in the adult brain and under what conditions RA is generated, and then genetically knockout RA-generating enzymes to remove endogenous RA and determine if this has an effect on synaptic plasticity. If so, then it would be relevant to determine whether the mechanism proceeds through nuclear RA receptors or in a nongenomic manner that involves cytoplasmic RA receptors or some other process. This is important as there currently are no RA genetic loss of function studies that support any nongenomic mechanism for RA.

## Data Availability

No data is provided in this report
